# Impact of Green Space, Air Pollution, and Weather on Cognitive Function in Middle and Old Age in China

**DOI:** 10.1093/geroni/igab046.2356

**Published:** 2021-12-17

**Authors:** Lingling Zhang, Ye Luo, Xi Pan, Qing Wang

**Affiliations:** University of Massachusetts Boston, Boston, Massachusetts, United States; Clemson University, Central, South Carolina, United States; Texas State University, San Marcos, Texas, United States; Shandong University, Shandong University,Jinan, Shandong, China (People's Republic)

## Abstract

Population aging will inevitably bring an increasing burden of poor cognitive function. The risk factors for cognitive decline have been widely studied. Even though environmental hazards have the greatest adverse impacts on older adults and the existing evidence has shown that green space, air pollution, and weather have an impact on cognitive function, most of the studies were conducted in developed countries and limited to cross-sectional analyses. China has the largest aging population in the world so the research evidence from it can offer an insight to the study in other developing countries facing similar issues and inform future public health policy and disease control. Using the data from a nationally representative sample of adults aged 45 years and older from the three waves of China Health and Retirement Longitudinal Study (CHARLS 2011-2015) and China City Statistical Yearbook, this study estimated multilevel growth curve models for the effects of green space coverage, air pollution, and weather conditions on cognitive function and cognitive decline. It showed that after controlling for sociodemographic characteristics, built area green coverage rate was positively associated with cognition score at baseline, and higher annual minimum temperature was associated with faster decline in cognitive function. These effects did not substantially change after weekly total hours of physical activities and levels of social engagement were added and the interaction effects were examined between environmental conditions with them, respectively. More research on the mechanisms of the effects of environmental factors on cognition is needed such as the subgroup analyses.

